# Impact of different methods of physical training in inflammatory cytokines of type 2 diabetes

**DOI:** 10.1186/1758-5996-7-S1-A229

**Published:** 2015-11-11

**Authors:** Pedro Weldes da Silva Cruz, Laisa Kalil Buarque, Denise Maria Martins Vancea, Moacir de Novaes Lima Ferreira

**Affiliations:** 1 Faculdade de Ciências Médicas de Pernambuco, Recife, Brazil

## Background

Changes in the levels of inflammatory cytokines such as tumor necrosis factor (TNF-α) and adiponectin may contribute to the aggravation of inflammatory processes the incidence increasing in 55% of cardiovascular events in diabetic patients. Exercise is indicated as part of diabetes treatment. According to the American Diabetes Association, various kinds of methods should compose a physical training program for diabetics, but the most used protocols are the aerobic and resistance, with fiw protocols that use the method of combined training.

## Objective

To identify which training method is more effective in relation to levels of proinflammatory cytokines and anti – inflammatory type 2 diabetics.

## Materials and methods

Study experimental nondandomized was approved by the Ethics Committee in Research of the Hospital Complex HUOC-PROCAPE/UPE on CAAE: 0154.0.106.000.11. 30 individuals with T2D were recruited who are part of the Sweet Life program Supervised Exercise Program for Diabetics the ESEF/UPE. The subjects were divided into 3 groups: Aerobic-GA n=10 which held 40 min. walk; Resisted-GR n=10 which held 8 strength exercises, and Combined -GC n=10 which held 20 min from GA and GR. The training program was performed 3 times a week for 24 weeks. The determination of cytokines (TNF-α and Adiponectin) was performed by Enzyme –Linked Immunosorbent Assay (ELISA). Analyzes of fasting glucose (8-12 h fasting). Blood postprandial glucose were also performed (after 1 hour of a standardized meal of 300 Kcal) made in the same intervals of cytokines by means of capillary glucose using Brezze2 glucometer from Bayer. Data were analyzed by non-parametric Wilcoxon test and Kruskal-Wallis beyond the Pearson Correlation, adopting a significance level of p ≤ 0.05.

## Results

The sample consisted mostly of women (n=25), mean age 66.4±8.7 yrs. When analyzing the impact of different training protocols on cytokines, the GR did not show changes in cytokine analyzed. In the intergroup analysis GA and GC showed a significant improvement in the values of TNF-α after the intervention (GA 12.7±1.32 vs 11.4±1.16 mg/mL p=0.001 and 13.7±GC 1.62 vs. 12.8±1.62 mg/mL p=0.000). There were no changes in adiponectin values after application of physical training protocol.

## Conclusion

The protocol of combined training showed greater efficiency in regulating the levels of TNF-α in this sample, with a greater emphasis on aerobic training.

**Figure 1:**
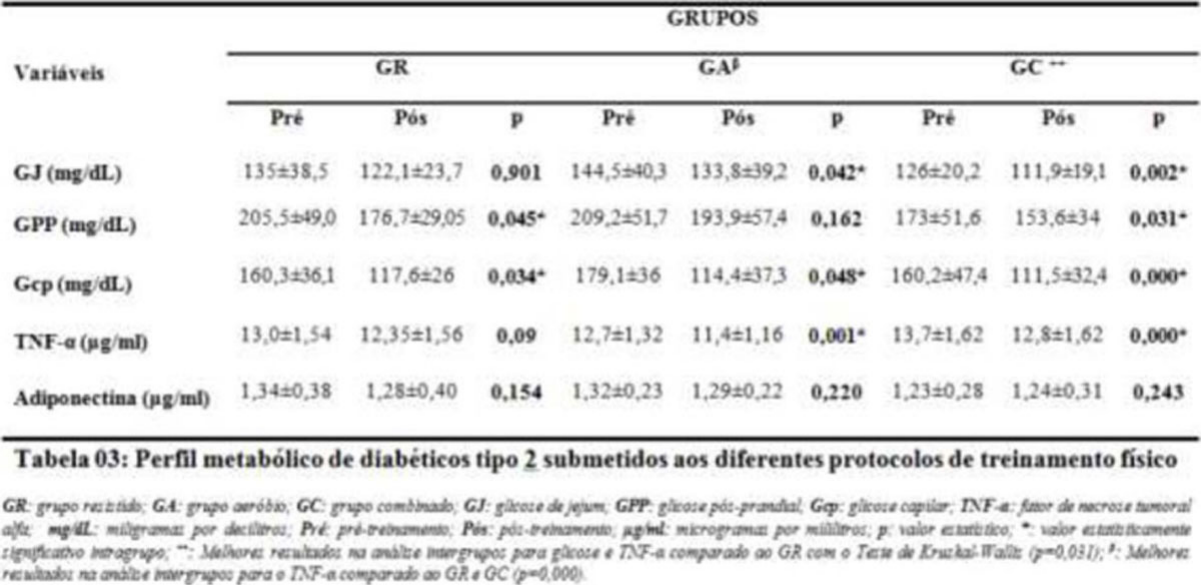
Metabolic profile of type 2 diabetics undergoing different types of training.

